# Hepatitis B knowledge among key stakeholders in Haimen City, China: Implications for addressing chronic HBV infection

**DOI:** 10.1186/s41124-016-0004-x

**Published:** 2016-04-14

**Authors:** Chari Cohen, Alison A. Evans, Peixin Huang, W. Thomas London, Joan M. Block, Gang Chen

**Affiliations:** 1 Hepatitis B Foundation, Doylestown, PA USA; 2 Drexel University School of Public Health, Philadelphia, PA USA; 3 Haimen City Center for Disease Control and Prevention, Haimen City, China; 4 Fox Chase Cancer Center, Philadelphia, PA USA

**Keywords:** Chronic hepatitis B infection, Hepatitis B knowledge and behaviors, Hepatocellular carcinoma, Gateway to Care campaign, Haimen City, China

## Abstract

**Background:**

This article describes hepatitis B-related knowledge, attitudes and practices after completion of the Gateway to Care campaign, a citywide public health education program that targeted city residents, health care providers and individuals chronically infected with hepatitis B virus in Haimen City, China.

**Methods:**

Pre/post questionnaires assessed hepatitis B knowledge change among health care providers and post-campaign surveys evaluated hepatitis B knowledge, attitudes and behaviors (including stigma-related beliefs and practices) among health care providers, city residents and chronically infected individuals. Focus groups were conducted to gain a more in-depth understanding of the needs of the target communities, and to identify future intervention strategies to improve hepatitis B testing and linkage to care and treatment.

**Results:**

Results indicate high levels of hepatitis B knowledge among multiple stakeholders in Haimen City, with significant knowledge improvement among health care providers. Stigma-related beliefs and myths regarding separation of infected individuals from certain aspects of family life were common among all stakeholder groups, despite high levels of accurate knowledge about hepatitis B transmission and prevention. Self-report of hepatitis B screening was low among city residents, as was awareness of hepatitis B treatment.

**Conclusions:**

More efforts are needed to improve awareness of HBV treatment, decrease HBV-related stigma, improve screening rates, and reduce cost of antiviral treatment. Future interventions in Haimen City should be driven by behavioral change theory, to not only improve knowledge, but to improve screening behaviors and address hepatitis B-related stigma and discrimination.

**Electronic supplementary material:**

The online version of this article (doi:10.1186/s41124-016-0004-x) contains supplementary material, which is available to authorized users.

## Background

An estimated 248 million individuals worldwide are chronically infected with the hepatitis B virus (HBV) [1]. Approximately 40 % live in China, where an estimated 100 million people are chronically infected, and where up to 500,000 people die annually from HBV-related complications, including primary liver cancer (hepatocellular carcinoma [HCC]) [2-5]. In China, most infection occurs at birth (perinatal transmission) or during early childhood, leading to high rates of chronic infection [4, 5]. The high prevalence of chronic HBV infection in China has yielded a high burden of HBV-related HCC incidence and mortality, with 55 % of liver cancer deaths worldwide occurring in China [6]. HBV immunization programs administered by the Chinese Ministry of Health and the World Health Organization (WHO), including the universal vaccination of newborns program begun in 1992, have improved HBV vaccine coverage in parts of China, which is reflected in a recent decline of prevalence rates [7, 8]. However, HBV vaccination does not help those who are already infected, and chronic HBV infection is still a considerable health issue in China with a serious public health impact.

The key to reducing morbidity and mortality associated with chronic HBV infection is improving rates of HBV screening and ensuring that infected individuals receive appropriate medical management [9, 10]. This is especially needed in areas that have high HBV prevalence and HCC incidence. However, multiple barriers result in low levels of screening, diagnosis, care and treatment for HBV. Key barriers to screening and vaccination include geography (i.e. rural areas), limited knowledge among the general population and health care providers, and HBV-related stigma [7, 9, 11, 12-14]. Treatment uptake is also low, primarily due to under-diagnosis and cost of antiviral treatment [15]. To help overcome barriers to HBV screening, the Hepatitis B Foundation (HBF) implemented the “Gateway to Care Campaign: Haimen City Project” in August 2010, in collaboration with the Haimen City Center for Disease Control and Prevention (HCCDC) [16]. This 3-year project is described in detail in a previous publication [16]. Briefly, the project consisted of a targeted citywide public health information and awareness campaign, specialized education and training for key constituencies (including health care providers, government officials and infected individuals), and expansions in health care infrastructure to increase screening, vaccination, treatment, and care management services.

This article describes HBV-related knowledge, attitudes and practices among key Haimen City constituencies (city residents, health care providers and infected individuals), as well as knowledge change among health care providers after implementation of the Gateway to Care program. The study also identifies potentially effective strategies that could be implemented in the future to improve HBV screening and care rates in Haimen City. Thus, these findings may guide the design of future interventions.

## Methods

### Haimen City, China

Haimen City, which has a population of 1.03 million, is located in Jiangsu Province, approximately 60 miles northwest of Shanghai. HCC has been the leading cause of cancer death in Haimen City since death registration began in 1970, and the incidence and mortality of HCC in Haimen City are among the highest in China, and in the world (Haimen City CDC Vital Statistics, unpublished data). City public health officials estimate that there are currently 80,000 residents aged 25 to 64 living with chronic HBV infection [16]. A population-based prospective cohort study enrolling more than 90,000 Haimen City residents found a 13.7 % chronic HBV infection rate. This study, which has been following infected individuals since 1992, established a direct link between viral load and liver cancer in chronically infected individuals [17]. The Gateway to Care campaign, designed by the HBF and HCCDC, grew out of the need for improved knowledge, screening and access to care in Haimen City. HBF, founded in 1991, is a United States-based non-profit organization engaged in scientific and public health research, education and patient advocacy.

### Gateway to care awareness and education program methodology

A multi-platform education and awareness campaign for the general public consisted of print media (newspapers, brochures, direct home mailings), community awareness events, and free educational giveaway products such as playing cards, drinking cups and calendars. All platforms provided information on HBV transmission, prevention, testing and treatment, as well as healthy lifestyle behaviors. In addition, educational messages highlighted the importance of being tested for HBV and the need for chronically infected individuals to receive regular check-ups to monitor liver disease progression. Another educational aim was to debunk common myths about HBV transmission, for example by explaining that HBV is not transmitted through casual contact or sharing eating utensils.

In addition to the public information and awareness campaign, 52 in-person educational seminars were provided for key constituencies. The seminars targeted citywide township government officials, village community leaders, township and village doctors, and chronically infected HBV patients hospitalized in the Haimen City People’s Hospital. The seminars were led by trained educators from HCCDC and infectious disease specialists from the People’s Hospital. Each seminar lasted for at least 1 h and focused on increasing knowledge about HBV transmission, prevention, testing, and appropriate medical management and treatment (including screening for early detection of HCC). A primary goal of the seminars was to encourage appropriate testing and referral to care, as per published clinical guidelines [18]. More than 90 % of targeted health care providers (1441) attended these educational seminars, including medical doctors, obstetricians, nurse assistants, school nurses, and public health officials from the 23 townships and 239 villages that comprise Haimen City.

Program evaluation focused on HBV-related knowledge, attitudes and practices of health care providers, city residents, and infected individuals. Due to logistical constraints, government officials were not a focus of the evaluation. There were two components to the evaluation: an assessment of change in knowledge, attitudes and behaviors among health care providers who attended in-person educational seminars; and an assessment of post-campaign knowledge, attitudes and behaviors among random samples of health care providers, city residents and chronically infected individuals.

### Data collection and analysis

To assess changes in knowledge before and after in-person seminars, health care providers were asked to complete a seven-item self-administered pre/post questionnaire. The pre/post questionnaire measured baseline knowledge and knowledge change immediately after seminar attendance in the following HBV-related domains: transmission, prevention, diagnosis, symptomology, epidemiology and treatment (Table 1, Additional file 1). A total of 710 providers completed the pre-questionnaire and 680 completed the post-questionnaire. To protect privacy and encourage participation, the pre- and post-questionnaires for individual participants were not linked. Results were analyzed using SAS v. 9.3 (Cary, NC). Poisson regression was used to examine stratum-specific differences in frequency of correct answers to the pre- and post-questionnaires by sex, age group, and educational level.

**Table 1:** Domains and variables of self-administered questionnaires

Domains of pre/post health care provider survey	HBV knowledge
	• Transmission • Prevention • Diagnosis • Symptomology • Epidemiology (local, national) • Treatment (when to treat, how to treat)
Domains of post-campaign self-administered surveys for health care providers, residents, and HBV-infected individuals	HBV knowledge
	• Transmission • Prevention • Diagnosis • Treatment
	Attitudes towards HBV
	• Transmission • Vaccination • Treatment • Importance of seeing a doctor • Importance of being tested
	HBV-related behaviors
	• Seeking HBV knowledge • Distancing from an infected individual • Getting tested for HBV • Getting vaccinated against HBV • Seeing a doctor if infected

Additionally, a post-campaign survey was conducted among random samples of health care providers (*n* = 227), Haimen City residents (*n* = 249), and chronically infected individuals (*n* = 20). These self-administered paper surveys evaluated HBV-related knowledge, attitudes and behaviors, including stigma-related beliefs and practices (Table 1, Additional file 1). Health care provider survey participants were recruited throughout the city, and city residents were recruited from two randomly selected villages. Chronically HBV-infected individuals were recruited from the Haimen City People’s Hospital. The surveys were analyzed using Microsoft Excel to calculate the percentage of correct responses on the HBV knowledge questions and the percentages of similar responses, by group, on the attitude and practice questions.

Six focus groups were conducted to gain a more in-depth understanding of how well the Gateway to Care campaign had met the needs of the target communities and to identify future intervention strategies for improving HBV testing, care and treatment. Focus groups were led by trained facilitators from HCCDC, and participants were recruited by HCCDC project staff.

Two focus groups were conducted with a total of 14 health care providers from two randomly selected townships. The providers were asked to comment on their preferred methods for receiving HBV education, strategies for improving HBV testing among community members, and strategies for promoting routine check-ups and antiviral treatment for chronically HBV-infected individuals. Two focus groups were conducted with a total of 15 city residents from two villages. These focus groups addressed methods for improving HBV awareness and HBV screening at the community level. Two focus groups were conducted with a total of 20 chronic HBV patients at the People’s Hospital, with patients asked for their perspectives on strategies for encouraging completion of check-ups and uptake of oral antiviral treatment. All focus groups were conducted in Chinese. All sessions were recorded, and notes were taken by the facilitators. Transcripts were translated into English for analysis.

Focus group data were analyzed by study team members from both HBF and HCCDC, using thematic analysis methodology as a guide to in-depth engagement with the text [19]. The process included multiple readings of the focus group transcripts to become familiar with the domains and themes of the text. A coding table was created to separate questions and responses into distinct domains for each of the three types of participants (providers, city residents and HBV-infected individuals) (Table 2). Coding of the text took place in two phases: open and selective coding. Open coding helped to identify themes and summarize the data generally. Selective coding was then used to code data related to the identified domains/variables. Memo-writing was used throughout the coding process to highlight key themes and relationships that were being identified within the text.

**Table 2:** Domains and primary questions for focus groups conducted with health care providers (*n* = 14), Haimen City residents (*n* = 15) and chronically HBV-infected individuals (*n* = 20)

Domains	Primary Questions
HBV knowledge	Providers and City Residents
Effective/preferred means of gaining knowledge	• What is the most effective way to gain knowledge about prevention and control of hepatitis B and liver cancer? • What is the best way for you to receive health education and promotion?
	Infected Individuals
	• What is the most effective way to gain knowledge about prevention and control of hepatitis B and liver cancer?
Attitudes towards HBV	Providers and City Residents
Necessary tools to fight the hepatitis B epidemic in Haimen City	• What is the most important help the community and patients need in fighting hepatitis B?
	Infected Individuals
	• What is the most important help you need in addressing hepatitis B?
HBV-related behaviors	Providers and City Residents
• Effective ways to encourage routine check-ups for infected individuals • Effective ways to encourage antiviral treatment for infected individuals who are candidates	• What is the best way to encourage hepatitis B testing for people unaware of their infection status? • What is the best way to encourage routine check-ups for asymptomatic hepatitis B patients? • What is the best way to encourage asymptomatic hepatitis B treatment candidates to receive appropriate antiviral treatment?
	Infected Individuals
	• What is the best way to encourage routine check-ups for asymptomatic hepatitis B patients? • What is the best way to encourage asymptomatic hepatitis B treatment candidates to receive appropriate antiviral treatment?

### Data anonymity and ethics statement

The program that this study is based on was reviewed and approved in 2010 by the Medical Ethics Review Committee of Haimen City. All data from the surveys and focus groups were collected anonymously, with no identifying information recorded.

## Results

### HBV-related knowledge change among health care providers who attended seminars

A total of 710 health care providers completed the HBV knowledge pre-test, 50.8 % of whom were male. The median age of the pre-test group was 45 (range 24–68). A total of 680 providers completed the HBV knowledge post-test, of whom 51.2 % were male. The median age of the post-test group was 46 (range 24–68). Neither age nor gender differed significantly between the pre- and post-test groups.

Respondents correctly answered 67.3 % of the pre-test questions and 85.9 % of the post-test questions, indicating an overall knowledge increase of 18.6 % (*p* < 0.0001) (Table 3). Male providers correctly answered 68.1 % of the pre-test questions and 85.6 % of the post-test questions, indicating a knowledge increase of 17.4 % (*p* < 0.0001) (Table 4). Female providers correctly answered 66.4 % of the pre-test questions and 86.3 % of the post-test questions, indicating a knowledge increase of 19.9 % (*p* < 0.01). There were significant knowledge increases for all age groups except the group aged 65 and older. Additionally, there were significant knowledge increases for those who had completed high school or less (14.4 %, *p* < 0.01) and those who had completed junior college (20.8 %, *p* < 0.0001). There was an 18.3 % knowledge increase among the small number of providers who had completed college (*p* = 0.06). Results of the Poisson regression indicate that controlling for age, gender and education (individually and together) did not change the estimate of the pre/post effect on HBV knowledge overall.

**Table 3:** Percent change in correct responses by health care providers completing pre/post HBV knowledge surveys

	Pre-Test	Post-Test	% Change	*P* value
	% Correct	% Correct		
Question 1	89.3	98.5	+9.2	<.0001
Question 2	79.2	94.4	+15.2	<.0001
Question 3	30.7	64.7	+34.0	<.0001
Question 4	58.5	74.1	+15.6	<.0001
Question 5	75.9	87.1	+11.2	<.0001
Question 6	65.5	87.8	+22.3	<.0001
Question 7	71.8	94.7	+22.9	<.0001

**Table 4:** Results of Poisson regression examining stratum-specific differences in frequency of correct answers to pre/post knowledge surveys among health care providers by sex, age group and educational level

Percent Correct by Group						Univariate	After Controlling for Pre/post Survey
	Pre-test (*N* = 710)	(*n*)	Post-test (*N* = 680)	(*n*)	Change	*p*-value	
Total	67.3 %	710	85.9 %	680	+18.6 %	<0.0001	
Gender							
Male	68.1 %	362	85.6 %	349	+17.4 %	<0.0001	0.24
Female	66.4 %	348	86.3 %	331	+19.9 %	<0.001	
Age Group							
< 35	66.9 %	117	87.6 %	107	+20.7 %	<0.001	0.13
35–39	61.4 %	144	85.3 %	131	+23.9 %	<0.001	
40–44	66.2 %	124	86.2 %	120	+20.0 %	<0.001	
45–49	68.6 %	66	87.3 %	63	+18.7 %	<0.01	
50–54	71.0 %	63	85.7 %	63	+14.7 %	0.05	
55–59	71.6 %	16	85.7 %	76	+14.1 %	0.03	
60–64	71.3 %	81	85.5 %	81	+14.3 %	0.03	
≥ 65	68.1 %	39	81.7 %	39	+13.6 %	0.17	
Educational Level							
≤ High School	70.5 %	213	84.9 %	213	+14.4 %	<.001	0.05
Jr/Tech Coll	65.1 %	429	85.9 %	408	+20.8 %	<.0001	
≥ College	71.0 %	68	89.3 %	59	+18.3 %	0.06	

### Post-campaign knowledge, attitudes and practices among health care providers

Of the 226 health care providers who completed the post-campaign questionnaire, 46.5 % were female and the mean age was 41 (age range 22–68). Nineteen percent had a high school education or less, 32.7 % had completed junior college, 20.8 % had completed college and 27.4 % had post-college secondary education. A total of 93.4 % of providers stated that they had received HBV education within the past 2 years. A total of 94.7 % correctly answered the questionnaire’s transmission question, 100 % correctly answered the HBV testing/diagnosis question, 88.5 % correctly answered the prevention question, and 93.8 % correctly answered the treatment question (Table 5).

**Table 5:** Percentages of HBV knowledge questions answered correctly in post-campaign surveys

	Health Care Providers	City Residents	Infected Individuals
HBV Transmission	94.7 %	85.9 %	95 %
HBV Diagnosis	100 %	84.3 %	100 %
HBV Prevention	88.5 %	80.7 %	95 %
HBV Treatment	93.8 %	57.8 %	80 %

Most providers (75.2 %) responded that it is necessary to test patients for HBV regardless of symptoms, while 24.3 % felt that it is important to test only patients who are symptomatic. Eighty percent reported that they tested their patients for HBV, 76.1 % reported suggesting to their patients that they be tested, and 85.8 % stated that they provide HBV education to their patients. Ninety-six percent of providers believed that infected individuals need regular check-ups, and 95.0 % advised their asymptomatic patients to get regular check-ups. While 97.8 % of providers agreed that it was important for treatment candidates to receive treatment, only 65.5 % of them reported suggesting treatment to their asymptomatic treatment candidates. Almost all providers (97.0 %) felt that individuals who are susceptible to HBV should be vaccinated, particularly if they have an infected family member, and 95.1 % reported recommending HBV vaccination to susceptible persons.

Health care providers participating in focus groups indicated that individualized, in-person, small group educational sessions were an effective way to acquire knowledge about HBV prevention and control. Providers also felt that a multi-platform strategy was the best way to improve HBV knowledge and awareness among members of the public. Suggested strategies for building public awareness included delivering HBV messages through mass media (potentially TV, newspapers and social media), community seminars, and in-person education by village doctors. Providers stressed the importance of repetitive messaging. Reducing the cost of laboratory tests was seen as the most important strategy for encouraging routine check-ups among HBV-infected individuals. Offering free treatment as well as educating infected patients about the consequences of HBV and about available treatment options were noted as ways to encourage treatment candidates to seek HBV treatment.

### Post-campaign knowledge, attitudes and practices among city residents

Of the 249 city residents who completed the post-campaign questionnaire, 55.8 % were female and the mean age was 48 (age range 18–88). Forty-nine percent had a middle-school education or less, 15.7 % had completed high school, 8.8 % had completed junior college or college, 2.0 % had post-secondary education, and 1.6 % had no formal education. About half (47.8 %) indicated that they had received some type of HBV education/information in the previous 2 years. A total of 85.9 % people correctly answered the questionnaire’s transmission question, 84.3 % correctly answered the HBV diagnosis question, 80.7 % correctly answered the prevention question, and 57.8 % correctly answered the treatment question (Table 5). Just over half of respondents (56.6 %) felt that it is either better, or necessary, for an HBV-infected individual to use separate utensils, and 24.9 % stated that they have tried to keep their distance from infected individuals because of fear of getting infected. The majority (67.5 %) believed that it is necessary to be tested for HBV, but only 35.3 % reported that they had ever been tested. Sixty-four percent of respondents felt that individuals who are susceptible to HBV should be vaccinated, and 42.2 % stated that they had been vaccinated. Eight percent did not know their HBV vaccination status. More than two-thirds of people (67.5 %) felt that infected individuals should have regular check-ups, and 75.9 % felt that treatment candidates should receive treatment even if they are asymptomatic.

The city residents who participated in the focus groups preferred to receive HBV health messaging through repetitive television seminars and face-to-face talks with their doctors. They believed that there was a need for people to have repeated exposure to HBV education rather than just hearing the messages once. They thought that improving HBV knowledge through education would encourage people to get tested, especially if free testing was available. Providing free or subsidized HBV treatment and educating people about the consequences of untreated HBV infection were thought to be effective strategies for encouraging infected individuals (including those who are asymptomatic) to seek treatment.

### Post-campaign knowledge, attitudes and practices among chronically infected individuals

Of the 20 HBV-infected individuals who completed the post-campaign questionnaire, 65.0 % were female and the mean age was 58 (age range 44–68). Thirty-five percent had completed elementary school, 55.0 % had completed middle school, and 10.0 % had completed high school. All (100 %) correctly answered the questionnaire’s HBV testing/diagnosis question, 88.5 % correctly answered the prevention question, and 93.8 % correctly answered the treatment question (Table 5). Almost all (95.0 %) correctly identified HBV transmission routes, while all (100.0 %) knew that a blood test was the only way to diagnose an HBV infection. Ninety-five percent were aware that the HBV vaccine could prevent transmission of HBV. Eighty percent knew that HBV-infected individuals might need treatment even if they had no symptoms. Eighty percent responded that they had received some type of HBV education in the past 2 years, and 95 % percent reported that they received a regular check-up at least once a year. All respondents stated that they had received HBV treatment at some point (55.0 % were on treatment and 45.0 % had taken treatment in the past). Thirty percent felt that it was necessary to separate their eating utensils from those of non-infected family members, and 65.0 % felt that while this was not necessary, it was advisable.

## Discussion

According to study results, HBV knowledge among health care providers significantly improved after attendance at an educational seminar. The results also indicate high levels of HBV knowledge among city residents and chronically infected individuals. However, city residents were less aware of the availability of treatment for HBV. For all three groups of study participants, having accurate knowledge was not necessarily enough to dispel stigma-related myths or foster behavior change regarding HBV screening or treatment. While 85.9 % of city residents correctly identified HBV transmission routes, more than half still felt that it was better to keep eating utensils of infected family members separate. One-quarter of these residents reported maintaining a distance from HBV-infected individuals for fear of becoming infected themselves. There were similar results among HBV-infected individuals who responded to the survey: virtually all correctly identified HBV transmission routes, but 65 % felt that it was better to not share their eating utensils with family members. Among providers, another gap between knowledge and practice was observed. While providers had high levels of knowledge regarding HBV transmission and diagnosis, almost 25 % reported that they only screen patients if they display symptoms of HBV. There also appeared to be discordance between providers and residents regarding testing, as only 35 % of residents recalled being tested for HBV.

While almost all providers agreed that it was important for treatment candidates to receive treatment, only 65.5 % of them suggested treatment to their asymptomatic treatment candidates. Results from the focus groups suggest that the high cost of antiviral treatment might play a role in this. Interestingly, among the 20 HBV-infected individuals who completed the post-campaign survey, almost all indicated that they underwent annual check-ups and had received treatment for their chronic HBV infection. However, it is not known if the respondents were referring to approved antiviral drug therapies for chronic HBV infection or to all types of treatment, including traditional Chinese medicine. The focus groups indicated that treatment was cost-prohibitive, and it has been documented that the proportion of HBV-infected individuals who receive antiviral treatment remains low in China [19]. More studies are needed in Haimen City to ascertain the percentage of infected individuals who routinely receive medical check-ups as per published guidelines and who receive appropriate antiviral treatment for their chronic HBV infection.

Additional research would be helpful in identifying strategies that could be used to enhance future educational/awareness interventions. Strategies should not only foster knowledge improvements, but also promote changes in beliefs and attitudes that can ultimately play a role in stigma reduction and behavior change. This could help to increase screening for all residents, improve treatment for some treatment candidates, and reduce discriminatory practices directed at infected individuals. Such strategies could be based upon behavioral change theories such as the Heath Belief Model, Health Behavior Framework and Transtheoretical Stages of Change [20]. These theories focus on multiple constructs that can be targeted for change, including knowledge, risk perception, beliefs, attitudes, and self-efficacy [20]. In doing so, they can guide the development of future interventions to improve effectiveness in changing HBV-related behaviors. Both the Health Belief Model and Transtheoretical Stages of Change theories have been used successfully to improve HBV screening behavior and similar types of cancer screening behaviors such as pap tests and colorectal screening [21, 14]. A study drawing on the constructs of the Health Behavior Framework looked at awareness of HBV, knowledge of transmission routes, perceived susceptibility, perceived severity, doctor recommendation, stigma of HBV infection, and perceived efficacy of testing among diverse ethnic groups in the United States (including Koreans, Vietnamese, Hmong, Chinese, Cambodians, and Taiwanese). Results indicate that it could prove to be a valid model for designing interventions aimed at overcoming challenges to HBV screening [22].

Stigma and discrimination surrounding hepatitis B in China and in other parts of the world has been well documented [23, 24, 11]. A study among Chinese Canadians found that perceptions of HBV-related stigma were associated with a decreased likelihood of undergoing HBV screening [11]. Strategies to overcome stigma have focused primarily on improving HBV-related knowledge, but little research has been done to assess the efficacy of this approach. A recent study conducted in rural China found that fear of HBV infection rather than lack of knowledge was what led to HBV-related stigma and discrimination [14]. Our results add evidence to the finding that knowledge by itself is not enough to overcome stigma and discrimination. Even with high levels of HBV knowledge, large proportions of those surveyed indicated a preference for separating themselves and/or their eating utensils from infected individuals, for fear of becoming infected. Studying this phenomenon further may lead to a better understanding of the pathway leading to HBV-related stigma and discrimination, which may in turn inform the design of appropriate theory-based interventions to reduce stigma and discrimination.

In terms of developing future HBV education and awareness efforts in China, focus group results across all groups of study participants suggest that repetitive educational interventions are advisable and that seminar-style education sessions should be supplemented with face-to-face (one-on-one) education by health care providers and with media-based communication. Additionally, since the costs of screening and treatment were thought to be prohibitive, future initiatives should address how these costs can be reduced.

It is necessary for key stakeholders, including health care providers, infected individuals and city residents, to have awareness and knowledge of HBV in order to address chronic HBV infection in China. Educational campaigns, such as the Gateway to Care, can play an important role. However, educational/awareness campaigns remain limited in their ability to improve HBV screening and care without seeing a change in access to HBV treatment. In China, cost seems to play a prohibitive role in both treatment recommendations by providers (as seen in this study) and treatment uptake by patients [19]. Improved access to treatment must be a necessary component in addressing chronic HBV infection in China into the future.

This study faced certain limitations. Pre-test data were not available from all groups, which limits the conclusions that can be drawn regarding the impact of the Gateway to Care campaign on knowledge change of city residents and chronically infected individuals. The pre- and post-intervention questionnaires measuring knowledge change among health care providers were not linked for individual study participants, so a matched analysis could not be conducted. A matched analysis might have yielded a better understanding of knowledge improvement as it relates to provider type or demographic factors. However, the pre-post analysis confirmed that the Gateway to Care campaign significantly improved HBV-related knowledge among participating providers who completed the pre/post surveys. Long-term follow-up was not completed, so we do not know if HBV-related knowledge change among providers was sustainable.

All questionnaires were specifically designed for use in this study, and while they were piloted tested, no formal psychometric testing was done to evaluate instrument validity and reliability (although low percentages of missing responses may indicate that questions were well understood by respondents). Additionally, while focus group participants were randomly selected, they are not necessarily representative of the greater population in Haimen City. This could limit the generalizability of conclusions that could be made from focus group data.

## Conclusions

The Gateway to Care education and awareness campaign was successful in fostering high levels of accurate HBV knowledge among health care providers in Haimen City, China. Additionally, city residents and chronically infected individuals were knowledgeable about HBV transmission, prevention and symptomology. City residents were less aware of the availability of treatment for chronic HBV infection, and results indicate that cost of treatment can serve as an additional barrier to care. Even with accurate HBV knowledge, all study groups indicated a preference for separating infected individuals in certain social situations. More research is needed to better understand and address the complexities associated with these stigma-related beliefs and practices. Future HBV programs in Haimen City should focus on reducing cost of antiviral treatment, and should continue to improve awareness and knowledge, but should also include the use of theoretically driven behavior change interventions to improve rates of HBV screening for city residents, as well as to increase the provision of appropriate care for infected individuals and treatment for treatment candidates.

## Additional file

Additional file 1: Healthcare provider pre/post-education questionnaire (knowledge questions only); and post-campaign questionnaire (knowledge questions only) for healthcare providers, city residents and chronically infected individuals. (DOCX 23 kb)

## Abbreviations

HBF: Hepatitis B Foundation
HBV: Hepatitis B virus
HCC: Hepatocellular carcinoma
