# Supplementary material for: Hepatitis B knowledge among key stakeholders in Haimen City, China: Implications for addressing chronic HBV infection
Source: Hepatol Med Policy. 2016 Apr 14;1:4. doi: 10.1186/s41124-016-0004-x (PMC5898514; doi:10.1186/s41124-016-0004-x)
Supplement: Supplementary file 1 — Healthcare provider pre/post-education questionnaire (knowledge questions only); and post-campaign questionnaire (knowledge questions only) for healthcare providers, city residents and chronically infected individuals. (DOCX 23 kb) [file 41124_2016_4_MOESM1_ESM.docx]

**Healthcare provider pre- and post-education questionnaire (knowledge questions only)**

1. Which of the following will not transmit hepatitis B?
2. From mother to newborn
3. Sharing utensils with hepatitis B patient
4. Through unprotected sexual contact
5. Contact with hepatitis B patient’s blood or infected body fluid
6. Don’t know
7. What is the most effective way to prevent hepatitis B infection?
8. Avoid contact with blood and body fluid from hepatitis B patient
9. Avoid contact with hepatitis B patient
10. Avoid sharing utensils with hepatitis B patient
11. Receive hepatitis B vaccine
12. Receive hepatitis B immunoglobulin
13. Don’t know
14. Which of the following hepatitis B markers can be used to confirm the hepatitis B diagnosis when it remains positive for more than 6 months?
15. Anti-HBs
16. Anti-HBc
17. Anti-HBe
18. HBeAg
19. HBsAg
20. Don’t know
21. Which of the following testing results indicate the need for hepatitis B vaccine to prevent new infection?
22. HBsAg (+) and anti-HBs (-)
23. HBsAg (-) and anti-HBs (+)
24. HBsAg (-) and anti-HBs (-)
25. HBsAg (+) and anti-HBs (+)
26. HBsAg (-) and anti-HBc (-)
27. Don’t know
28. Which of the following is incorrect?
29. In China chronic hepatitis B infection mainly occurs at birth or in childhood
30. Most hepatitis B infections at birth or in childhood have no symptoms
31. A person can be asymptomatic for a long time after hepatitis B infection
32. Chronic hepatitis B infected individuals don’t need to see a doctor if they have no symptoms
33. Don’t know
34. Which of the following is incorrect regarding the treatment of hepatitis B?
35. All chronic hepatitis B infected persons will need treatment
36. Long-term treatment under a doctor’s instruction is needed for the treatment of chronic hepatitis B
37. Appropriate treatment of chronic hepatitis B can prevent liver cancer
38. Currently there is no cure for chronic hepatitis B
39. Don’t know
40. Which of the following is the most effective treatment for chronic hepatitis B?
41. Anti-inflammatory drug
42. Anti-fibrosis drug
43. Anti-viral drug
44. Drug improving liver function
45. Chinese herbs
46. Don’t know

**Post-campaign questionnaire (knowledge questions only) for healthcare providers, city residents and chronically infected individuals**

1. Which of the following will not transmit hepatitis B?
2. From mother to newborn
3. Contact hepatitis B patient’s blood
4. Through sexual contact
5. Sharing utensils with hepatitis B patient
6. Which of the following can be used to confirm hepatitis B infection?
7. Has hepatitis B symptoms
8. Blood test
9. Image test
10. Physical exam
11. What is the most effective way to prevent hepatitis B infection?
12. Avoid contact with hepatitis B patient
13. Avoid sharing utensils with hepatitis B patient
14. Avoid contact with blood and body fluid from hepatitis B patient
15. Receive hepatitis B vaccine
16. Which of the following is incorrect regarding the treatment of hepatitis B?
17. Currently there is no cure for chronic hepatitis B
18. Not every infected person will need treatment
19. Chronic hepatitis B infected person does not need treatment if there is no symptom
20. Appropriate treatment of chronic hepatitis B can prevent liver cancer
